# Mission Architecture Using the SpaceX Starship Vehicle to Enable a Sustained Human Presence on Mars

**DOI:** 10.1089/space.2020.0058

**Published:** 2022-09-13

**Authors:** Jennifer L. Heldmann, Margarita M. Marinova, Darlene S.S. Lim, David Wilson, Peter Carrato, Keith Kennedy, Ann Esbeck, Tony Anthony Colaprete, Richard C. Elphic, Janine Captain, Kris Zacny, Leo Stolov, Boleslaw Mellerowicz, Joseph Palmowski, Ali M. Bramson, Nathaniel Putzig, Gareth Morgan, Hanna Sizemore, Josh Coyan

**Affiliations:** ^1^ Division of Space Sciences and Astrobiology, Planetary Systems Branch, NASA Ames Research Center, Moffett Field, California, USA.; ^2^ Independent Consultant, Santa Monica, California, USA.; ^3^ Bechtel Corporation, Reston, Virginia, USA.; ^4^ NASA Kennedy Space Center, Kennedy Space Center, Florida, USA.; ^5^ Honeybee Robotics, Pasadena, California, USA.; ^6^ Department of Earth, Atmospheric, and Planetary Science, Purdue University, West Lafayette, Indiana, USA.; ^7^ Planetary Science Institute, Tucson, Arizona, USA.; ^8^ United States Geological Survey (USGS), Geology, Minerals, Energy, and Geophysics Science Center, Spokane, Washington, USA.

**Keywords:** Mars, human exploration, ISRU, Starship, SpaceX

## Abstract

A main goal of human space exploration is to develop humanity into a multi-planet species where civilization extends beyond planet Earth. Establishing a self-sustaining human presence on Mars is key to achieving this goal. *In situ* resource utilization (ISRU) on Mars is a critical component to enabling humans on Mars to both establish long-term outposts and become self-reliant. This article focuses on a mission architecture using the SpaceX Starship as cargo and crew vehicles for the journey to Mars. The first Starships flown to Mars will be uncrewed and will provide unprecedented opportunities to deliver ∼100 metric tons of cargo to the martian surface per mission and conduct robotic precursor work to enable a sustained and self-reliant human presence on Mars. We propose that the highest priority activities for early uncrewed Starships include pre-placement of supplies, developing infrastructure, testing of key technologies, and conducting resource prospecting to map and characterize water ice for future ISRU purposes.

## Introduction

Space exploration as a collective has many arcs and interconnected goals such as to find life beyond Earth, to understand the formation of our Universe, and as discussed here, to evolve humankind into a multi-planet species.^1–3^ A foundational step in this journey is to develop a self-sustaining human civilization on Mars, which is the closest planet to Earth that is realistically capable of harboring human communities and cities.^1–7^ The pathway for sustained human Mars exploration includes (1) uncrewed landed missions to Mars, followed by (2) human landed missions to Mars with relatively small (10–20 person) crews to establish the first human presence on the planet, (3) advanced infrastructure development to support planned community growth, and then (4) transitioning to a self-sufficient state on Mars.

The fourth and final condition will require sustained *in situ* resource utilization (ISRU), which is the ability to survive on Mars using local resources and eliminating the reliance on Earth for long-term survival. Extraction and processing of critical *in situ* resources for use such as propellants, life support, power generation, and radiation and rocket exhaust shielding, among other applications, can significantly reduce the required launch mass, risk, and cost of human and robotic space exploration.^7–14^

The most valuable resource on Mars for ISRU is the vast deposits of martian water ice.^15–23^ Water will be required for uses such as life support and agriculture, and it will also be utilized through electrolysis to produce hydrogen and oxygen for use in fuel cells and rocket engines. Each kilogram of consumable material transported from Earth incurs a penalty of about 200 kg of propellant to launch, transfer, and land on Mars, depending on vehicle specifics. The ISRU of water ice facilitates the development of a self-sustaining civilization since the launch mass penalty for transporting water from Earth to Mars is eliminated.^24^

Because of the importance of martian water, the characterization of the ice resource is a top priority for near-term robotic flights to Mars in preparation for human exploration. This paper, thus, focuses on an assessment of initial uncrewed flights to Mars aimed at characterizing the ice resource availability plus the methods and equipment required to extract and process the ice to support future human exploration. In addition, sustained human travel to and survival on Mars is a challenging goal that will require expertise on various topics such as on-site construction, infrastructure planning and development, power systems, communications capabilities, and human health and safety considerations, etc., which must also be addressed as early as the first uncrewed missions.

**Fig. 1.**
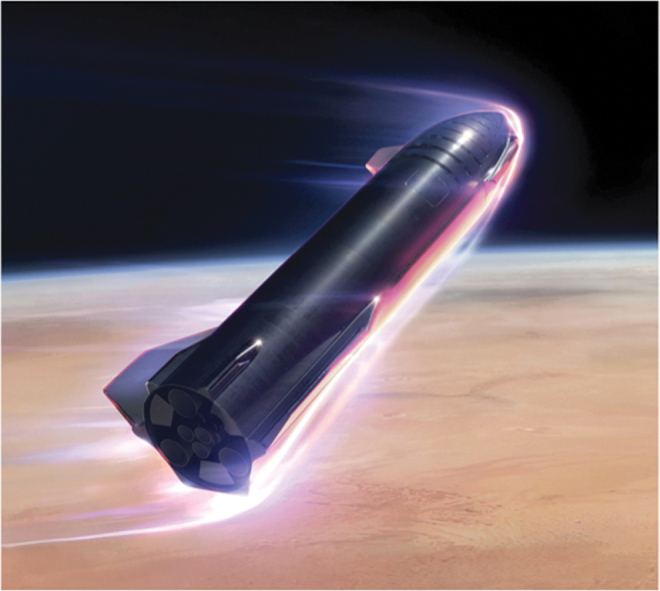
Artist rendering of the SpaceX Starship vehicle entering the martian atmosphere. Credit: SpaceX.

This article presents a mission architecture assuming use of the SpaceX Starship vehicle (*Fig. 1*) for all landed missions to accomplish the earlier stated goals and tasks. The first uncrewed Starships to land on Mars should be strategically dedicated to resource prospecting, infrastructure development, and technology demonstration before human arrival. These early missions can demonstrate the ability to land human-scale landers on Mars and provide the opportunity for the ground-truth of potential landing site(s) for the eventual human Mars base. The uncrewed Starship flights also provide the opportunity to test high-risk items that are critical to ISRU and long-term human settlement early, and they can autonomously construct basic infrastructure components such as roads, landing pads, and protective berms.

## Starship Mission Architecture

We consider a Mars mission architecture predicated on the SpaceX capabilities and vehicles. For the purposes of this article, we make the following assumptions regarding the SpaceX capabilities and outline a candidate mission architecture accordingly.

We assume that initially, at least two uncrewed Starships will be launched to Mars.^1^ These uncrewed vehicles have the capability of landing within close proximity of one another (∼1 km spacing to avoid damage induced from landing plumes and lofted regolith) at one landing site or can land in separate regions of Mars if *in situ* site reconnaissance is warranted in different locations to select the final landing site for the crewed missions. The arrival of Starships at Mars could also be staggered by ∼1–2 months such that landing site assessment can be performed with data returned from the first Starship before deciding on the final landing site for the subsequent Starship vehicle(s) during a given launch window. These first uncrewed Starships should remain on the surface of Mars indefinitely and serve as infrastructure for building up the human base.

Launch windows from Earth to Mars occur approximately every 26 months when the Earth and Mars are optimally aligned for interplanetary travel with maximum speed and minimal propulsion costs,^25–27^ and thus the next wave of Starships can be launched at the next launch opportunity. This second wave of launches would include two or more uncrewed vehicles plus at least two crewed Starships; all of these vehicles can land at the preferred landing site for construction of the human Mars base. Starship launches then continue at each subsequent launch opportunity. An optimal plan is for the total number of landed vehicles to double at a minimum with each consecutive opportunity, with a to-be-determined split between crewed and uncrewed vehicles. These landings should be focused at the site of the human Mars base.

### Starship Capabilities

Starship will be launched by a SpaceX Super Heavy Booster.^28,29^ This two-stage vehicle (Super Heavy first stage and Starship as the second stage) is fully reusable and can provide transport to the Earth orbit and the Moon, as well as Mars.^3,28,30^ Starship will serve as the lander for both uncrewed and crewed missions, customizing the payload volume depending on the mission.

Given its expected payload capacity, Starship is able to transport the necessary equipment to support sustained human exploration as well as the crews, enabling the eventual establishment of cities on Mars.^3^ Musk^1^ outlines how SpaceX missions to Mars will utilize in-space propellant transfer. In this case, the Booster launches Starship into the Earth orbit where it is refilled with CH_4_ and O_2_ by additional tanker flights from Earth (tankers are Starships that carry only propellant as payload); the Boosters and tankers return to the launch site for reuse. The refilled Starship vehicle then travels to the surface of Mars.^28^ Refilling Starship in orbit effectively resets the rocket equation, allowing for large payloads to be transported to the Moon and Mars.

We utilize the expected capabilities and performance of Starship for this analysis. Starship will be capable of delivering 100 metric tons of payload to the martian surface^3^ and can utilize both forward and aft storage capacity.^30^ Starship is 9 m in diameter and 50 m in length. Nominal payload deployment doors are assumed to be 3 m × 3 m and can be further customized if necessary for specific payloads. Initial payloads will require significant autonomy for deployment to the surface and operations, whereas future payloads will have more crew oversight once a human presence has been established on the planet.

Starship is also capable of returning crew and cargo from Mars to Earth. The vehicle is refilled with propellants on Mars by using local resources processed through a surface propellant production plant.^3^ Starship then launches from Mars and conducts a direct return to Earth.^28^

### Starship Human Flights

Starship flights carrying the first humans to Mars are optimally planned for the Mars launch window after the launch of the first two (or more) uncrewed Starship vehicles. Therefore, on human arrival on Mars, there will already be at least two cargo Starships on the surface. This second wave of missions can include two Starships carrying crew plus additional uncrewed/cargo Starships. The human Starships will have on order of 1,100 m^3^ forward space, most of which will be pressurized for human habitation,^28,30^ an 800 m^3^ LOX tank, and a 600 m^3^ methane tank with a stainless steel primary structure.

The LOX and methane tanks could later become pressurized living space on the surface of Mars. We recommend that these first crewed Starships each have about 10–20 total people onboard with an additional 100+ metric tons of available cargo mass per Starship. Cargo carried on these flights will necessarily include additional equipment required for human health and productivity during the transit to Mars and on the martian surface. These vehicles will also carry fully operational hardware needed to support the human Mars base, which is likely to include equipment for power production, water extraction, pre-prepared landing pads, radiation shielding, dust control equipment, and exterior shelters for humans and equipment.

Humans will likely live on Starship for the first few years on Mars until additional habitats are constructed, so the radiation risk must be assessed and mitigated accordingly and equipment planned to support this initial infrastructure. The first wave of uncrewed Starship vehicles can also be relocated and/or repurposed as needed to support the people on the surface. These vehicles will be valuable assets for storage, habitation, and as a source of refined metal and components.

## *In Situ* Resource Utilization

As a sustained presence of humans on Mars is predicated on ISRU, a key objective for the first uncrewed Starship missions is to confirm the presence of water ice (and other desired resources) and characterize these resource deposits. This work would serve to either (1) validate the selection of the initial landing site as satisfactory for subsequent human landing or (2) provide valuable information to consider moving the human landing site to a different location. Here, we describe multiple uses of water for ISRU and a suggested payload for characterizing the distribution and properties of near-surface water ice for ISRU.

### Utility of Water Ice as a Mars Resource

An estimate of requirements for water consumption in the first 5 to 7 years of human habitation is needed to create a notional water budget for an initial Mars base. Based on space station experience, the amount of water required (without recycling) is estimated at 0.6 kg/h/person, which includes water for consumption, hygiene, and everyday living.^31–34^ This estimate will be higher for a longer-term base on Mars with fewer restrictions on water use such as using more water for personal hygiene compared with current International Space Station (ISS) protocols, and/or allowing more water for activities such as laundry and cleaning dishes.

The amount of water required per person will also increase as the Mars community grows and eventually includes activities that use water in addition to personal hygiene and hydration (*e.g.*, regolith processing, manufacturing, construction, perchlorate remediation, plant growth, habitat maintenance, etc). When designing water treatment and distribution systems for Mars, it is important to recognize that there will be various uses of water, which will each have different requirements for purity (*e.g.*, water for human consumption vs. water for construction).

In addition to supporting human survival, water will be used as a main source of propellant production on Mars. Propellant will primarily be used to allow Starship vehicles for return to Earth with both cargo and crew. Starship has an oxidizer to fuel (O/F) ratio of ∼3.5. With its 1,200 metric ton propellant capacity, Starship requires on order of 933 metric tons of oxygen and 267 metric tons of methane for refilling on Mars. Through the use of water electrolysis and Sabatier reactions, the relevant net ISRU reaction to produce this oxygen and methane is CO_2_ + 2H_2_O → CH_4_ + 2O_2_.

This net reaction produces a mass ratio of O/F of 4 and therefore excess oxygen will be produced through this process, which can be used for breathing. The total quantity of water needed to refill one Starship through these processes is then on the order of 600 metric tons, and equivalent to an ice cube about 9 m on the side.

There are multiple options for obtaining this water to support return flights to Earth for the first few crews on Mars. Water can be transported to Mars from Earth within Starship cargo vehicles to ensure the availability of necessary water for life support and propellant production. This approach will be especially important for ensuring life support needs for the first few human crews on Mars, before ISRU of water ice is fully functional and reliable.

Bringing water from the Earth for propellant production is also likely possible in an emergency situation but is challenging, requiring the expensive delivery of water from Earth. Ultimately, the required water will be mined as a natural resource on Mars to support the expanding base and provide propellant for routine return trips to the Earth.

### Accessibility of Water Ice on Mars

As a resource required for survival and growth of a human civilization on Mars, the location and ease of access of water ice are key drivers for the landing site selection. The location of the Mars base, adjacent to the landing site, is one of the most critical decisions to be made as all other mission architecture planning and trade studies must be copacetic with the characteristics of this site.

An architecture to characterize the ice *in situ* at and near the first uncrewed Starship landing sites must, therefore, be developed. This on-the-ground reconnaissance is critical, because we must validate assumptions based on orbital and remote-sensing data, coupled with geological modeling. A top reason for characterizing the near-surface ice is to inform the development of ISRU systems. Knowing the form and purity of the ice (*e.g.*, massive ice, pore ice, etc.) as well as its distribution (depth to the ice, and the lateral and vertical concentrations/heterogeneities) is important for planning the ice excavation operations and ISRU processing.

Understanding the distribution and quantity of the ice in the vicinity of the landing location may be especially important, as orbital observations have relatively large footprints, often averaging data over many square kilometers, and local variations may be significant. These uncrewed reconnaissance missions will not only demonstrate whether it is safe and how to land Starships at the site, but also provide data to determine whether there is, indeed, a viable ice resource in the vicinity of the landing site.

### Ice Resource Characterization

Characterization of the near-surface ice resource will be accomplished by analyzing data collected using robots and rovers delivered by the first uncrewed Starship missions. This *in situ* characterization will provide the ground truth needed to assess the viability of the ice resource at the chosen landing site and inform the final decision for the human landing location on Mars.

A number of *in situ* measurements are required to characterize the ice resource. In addition to the composition, distribution, and form of the ice, the deposit's overburden must be understood. Depth and material properties of the overburden will influence the design and operation of the ISRU system needed to penetrate to the ice itself. Local geology and trafficability of the site are also important for general site knowledge and for planning of larger-scale ISRU processing and resource transportation systems.

To accomplish these objectives, a planetary instrument suite is proposed that has already been selected for flight to the Moon by NASA. The VIPER (Volatiles Investigating Polar Exploration Rover) mission is slated for flight in the mid-2020s to characterize the lateral and vertical distribution of volatiles on the Moon with the goal of helping to inform lunar ISRU architectures.^35^

Although orbital observations of volatiles provide valuable information, missions such as VIPER will provide measurements on centimeter-scale resolution over distances of kilometers that are needed to generate adequate “volatile mineral models” for use in evaluating lunar resource potential.^35^ The situation is similar for Mars, in that orbital data coupled with numerical modeling are suggestive of near-surface ice, but *in situ* data at higher confidence and at finer spatial scales are important to verify the potential for ISRU and required for optimizing the engineering solution for obtaining the ice.

**Fig. 2.**
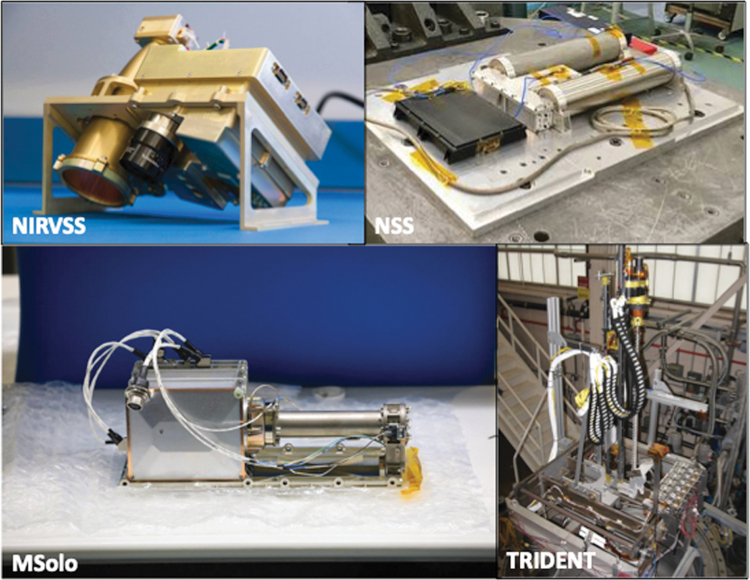
NASA's VIPER payload: NIRVSS (*top left*, photo credit NASA), NSS (*top right*, photo credit NASA), MSolo (*bottom left*, photo credit NASA/Glenn Benson), and TRIDENT (*bottom right*, photo credit Honeybee Robotics/Kris Zacny). NIRVSS, Near-InfraRed Volatiles Spectrometer System; NSS, Neutron Spectrometer System; TRIDENT, The Regolith and Ice Drill for Exploring New Terrain; MSolo, Mass Spectrometer Observing Lunar Operations.

The VIPER payload recommended for flight to Mars consists of several instruments integrated with a mobility platform (lateral motion) and subsurface drill (vertical access) to characterize near-surface ice (*Fig. 2*). The payload includes a neutron spectrometer, near-infrared spectrometer, mass spectrometer, and drill subsystem to map and characterize water ice.^36^ Each payload element is discussed here.

#### Neutron Spectrometer System

Neutron spectroscopy has been used at multiple solar system targets to measure planetary bulk composition and hydrogenous volatile abundance. The Neutron Spectrometer System (NSS) provides (1) estimated hydrogen abundance via epithermal neutron flux, and (2) bulk regolith chemical information via thermal-to-epithermal neutron flux.^36^ NSS is, therefore, a key tool to map buried ice.^37^

The NSS measures both thermal and epithermal neutrons and is active during roving and drilling operations. The instrument operates by measuring the changes in the leakage flux of low energy neutrons out of the regolith. These neutrons are produced by galactic cosmic rays, which are so energetic that they shatter the nuclei in surface materials. The neutrons interact with other nuclei and lose energy, becoming thermalized in the process.

Hydrogen is the most efficient at thermalizing neutrons because of their similarity in mass to protons. Water will necessarily have a hydrogen signature, though a hydrogen signal by itself does not uniquely identify the presence of water.

#### Near-InfraRed Volatiles Spectrometer System

The Near-InfraRed Volatiles Spectrometer System (NIRVSS) measures volatile composition, mineralogy, thermophysical properties, and fine-scale geomorphology.^38^ The instrument operates both by viewing the surface underneath a rover while driving and also by viewing subsurface drill samples. Using different wavelengths of light to illuminate the surface, NIRVSS surveys the surface and excavation site for water and other volatiles, providing surface and regolith mineral context.

#### Mass Spectrometer Observing Lunar Operations

The Mass Spectrometer Observing Lunar Operations (MSolo) instrument is a modified commercial off-the-shelf instrument based on INFICON's Transpector^®^ MPH high-performance quadrupole mass spectrometer. The instrument analyzes volatiles released from the regolith during traverses as well as subsurface regolith extraction via drilling. MSolo can detect and differentiate low-molecular-weight volatiles between 1 and 100 atomic mass units. This capability allows for the estimation of water (H_2_O) abundance, and the identification of and relative intensities of various volatile species (including H_2_, He, CO, CO_2_, CH_4_, NH_3_, H_2_S, SO_2_, etc.).

#### The Regolith and Ice Drill for Exploring New Terrain

The Regolith and Ice Drill for Exploring New Terrain (TRIDENT) is a 1-m rotary-percussive drill developed by Honeybee Robotics and designed to deliver dry and volatile-rich regolith to the surface for inspection by NIRVSS and MSolo. TRIDENT relies on a full-faced drill bit with carbide cutters at the end of a 1 m auger to break up regolith and convey cuttings to the surface. The auger is split into two sections: The bottom 10 cm (also referred to as a “sampling bit”) has deep flutes at shallow angle which are ideal for retention of material; the upper 90 cm section has shallow flutes at steep angle which are suited for efficient material removal.

This design allows for a “bite sampling” approach that preserves subsurface stratigraphy. To reduce drilling power, TRIDENT delivers subsurface regolith in ten 10 cm “bites” all the way to 1 m depth (or deeper depending on drill string length).^39,40^

To detect deeper ice within the upper few 10s of meters of the martian surface, a geophysics payload may also be considered. Shallower ice is preferred for ease of access to the resource, whereas deeper ice may still be valuable if it has higher purity or other beneficial characteristics. In all cases, also understanding the ice characteristics with depth and the total ice thickness is important for resource utilization planning. Multiple geophysical techniques are used terrestrially to detect subsurface ice, including ground penetrating radar, DC resistivity sounding, seismic refraction, and electromagnetic surveys.^41,42^ Both surface and airborne deployed instrumentation can be considered depending on the trafficability of the landing site area on Mars.

Mobility systems are key to enabling these measurements of ice distribution. There are two different types of potential ice-rich landing sites on Mars for Starship: (1) subsurface ice in the northern hemisphere plains and (2) lobate debris aprons (LDAs), or rock glaciers with ice buried beneath a surface layer of rock and debris.^43^ Each type of site likely requires a different style of mobility platform. The northern plains ice could easily be trafficked by a typical rover given the relatively flat terrain and ability to navigate around any local obstacles.

Data collection on an LDA is likely more complex given the complicated topography often associated with these geologic features. Unless a particularly smooth area with suitable geotechnical properties is found, a typical wheeled rover likely could not traverse across an LDA given the expected instability of the surface and the sizes and frequency of rocks and boulders. In this case, airborne assets, vehicles with enhanced mobility capabilities, and/or instrumented impactors may be required to access and measure the LDA.

The current state of the LDA (*e.g.*, if the LDA is actively flowing or not) will also affect the location of maximum ice concentration (*e.g.*, near the head or base of the glacier). An option is to access ice horizontally along the glacier margins (instead of from the top of the LDA) if the accumulation of rocks is not prohibitive near the toe or margins of the glacier. Before investigating an LDA, additional site selection work would be required to determine the local trafficability and site access as well as to identify locations within the LDA with the highest probability of hosting accessible ice.

Ideally, multiple mobile platforms would be deployed on Mars to cover more ground in less time, facilitating a more comprehensive study of the ice distribution in the vicinity of the Starship landing site. Given the large payload mass of Starship, multiple instrumented mobile assets can be sent and deployed. This architecture changes the risk posture of the current NASA paradigm of typically sending one mobile asset at a time to Mars (Mars Science Laboratory, Mars Perseverance).

The mobility vehicles can be made robust and able to navigate significant terrain challenges since mass need not be minimized given Starship's large payload capability. Easy access from the landed vehicle to the martian surface is possible from within the payload pods at the base of Starship. On landing, these vehicles can be deployed directly on the surface without the need for complex unfolding or deployment activities as have been used on previous missions.

Additional vehicles can also be stowed and deployed from the main (forward) cargo area. The vehicles must be robust enough to traverse the martian terrain near the landing site but should not be overdesigned or optimized at high cost since more risk tolerance is allowed by deploying many assets at once. If one or several vehicles are lost or malfunction, the remaining fleet can accomplish the task of mapping the water ice. For the relatively benign topography of the plains ice site with its easy trafficability, vehicles similar to those used in many terrestrial environments could serve as mobility platforms to characterize the subsurface ice.

To understand the resource potential of the martian ice, the spatially distributed resource prospecting data must be synthesized to determine optimal locations for resource extraction and utilization. Resource assessments are common practice in terrestrial mining operations and provide a framework for making decisions under conditions of uncertainty by first filling in data at locations that were not sampled and then supplying information about resources in terms of potential occurrence, distribution, type, quality, amount, value, and certainty in assessment results.^44^

Mineral Prospectivity Mapping (MPM) is a geospatial mathematical technique for predicting the location and likelihood for the presence or absence of a mineral (or resource) accumulation. The result of mineral potential modeling is a collection of prospectivity maps highlighting areas as not-permissive, permissive, favorable, and prospective (*i.e.*, areas that are the most likely to contain, in this case, concentrations of water ice).^45^ The ability to geostatistically analyze ice prospecting datasets, determine cross-correlation relationships among *in situ* remote sensing datasets, and create predictive maps regarding water ice for ISRU (including areas where ground-truth data are not available) will be critical components of the prospecting process.

### Considerations for ISRU Water Ice System Criticality

The use of water ice for ISRU has been determined as a critical feature of sustainability for a long-term human presence on Mars. With this goal in mind, early planning must differentiate between critical systems for enabling a long-term human presence versus supplementary capabilities, with an emphasis correspondingly placed on the critical systems during early mission opportunities. For example, infrastructure must be built to be expandable to support additional facilities over time.

Tools and equipment used within the base should be multi-purpose whenever possible to maximize operational flexibility and optimize the mass allocations that are transported from the Earth. Multiple capabilities for equipment and tools will also ensure redundancy and resilience. For example, water extraction should employ multiple systems to ensure that if one method fails, the crew will not be jeopardized. As the size of the Mars base expands, we must also remain mindful of complexity growth. Multiple approaches for initial activities and installations will provide additional capacity and faster growth potential for the future. The need for spare parts must also be addressed, especially as there will likely be limited knowledge on the durability of these systems in the martian environments. Using common components between systems will aid in having sufficient spare parts while also minimizing the mass required from Earth.

### Water Ice ISRU Process

Water ice ISRU on Mars is based on terrestrial mining processes and modified for the martian case. The water systems planning is critical, because a robust water supply and wastewater treatment system is fundamental to establishing and developing an urban environment, and as such will be required for the growth of a human base on Mars. Thus, the initial system layout and design should be conducted with a long-term design in mind. Here, we divide the ISRU process into five steps and describe the necessary activities to execute each stage of the ISRU sequence.^46^

#### (1) Characterization of ice reservoir (resource exploration/prospecting)

The first step to enable ISRU is to characterize and verify the presence of the resource. The distribution (vertical and horizontal) of the potential reserve must be mapped, and the form and concentration of the resource (ice) in the subsurface must be determined. Surface ice is largely not stable in the mid to low latitudes on the surface of Mars in the present-day climate,^19,47^ and thus any ice will have an overburden layer of dry rock and/or regolith. This overburden depth must be determined, along with the material strength and properties (*e.g.*, rock, loose soil) of the layer to ensure suitable drilling and/or excavation techniques are used to access the buried ice.

Understanding the local geologic setting is also important for characterizing the subsurface ice itself as well as the overburden properties. Understanding the local geology and history of the site will inform the type of ice present in the subsurface (*e.g.*, pore ice vapor deposited from the atmosphere, massive ice remnant from a relict glacier, frozen flood waters or snowpacks). Depending on the site, the overburden material may be wind-blown sands and fines, regolith and/or rock resultant from ice sublimation over geologic time, glacial depositions of larger rocks and boulders, headwall erosion, etc.

#### (2) Acquisition of water ice

There are multiple techniques for acquisition of subsurface ice on Mars. The Rodriguez well (Rodwell) technology is a terrestrial method for extracting water in polar environments that is promising for martian application. The Rodwell was first designed and tested for use in Greenland and Antarctica.^48,49^ The concept is straightforward and involves melting ice at depth to create a reservoir of liquid water that can be pumped to the surface. The development of the size and shape of the underground ponding cavity are functions of the relative rates of melting and water removal via pumping.^50^ The Rodwell maintains liquid water within the subsurface cavity for the duration of the well's operational lifespan, and this pool of water expands as more ice is melted over time from the walls of the cavity, thereby providing a constantly renewing source of water.

Rodwell systems are robust and still in routine use in polar regions on Earth. A Rodwell has been used at the U.S. South Pole Station in Antarctica since 1995 to supply liquid water to the station^51^ and has successfully provided tens of millions of liters of fresh water.^52^ The lifespan of a South Pole Rodwell is ∼7 years, which would be ideal for Mars since this timeframe would cover multiple launch opportunities to support an initial base of humans.

**Fig. 3.**
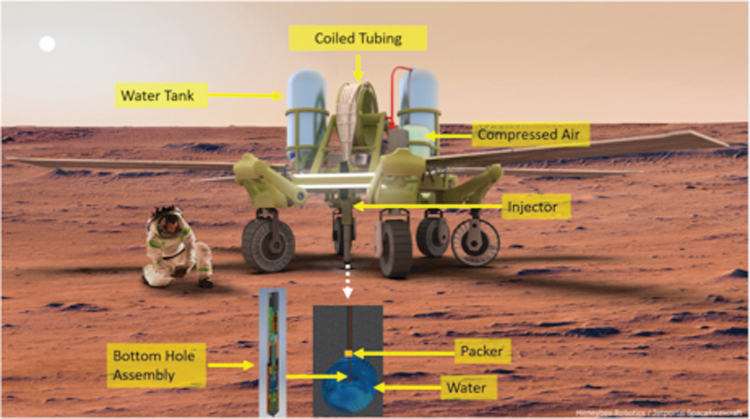
Schematic diagram of Honeybee Robotics RedWater Rodriguez well for Mars. Major subsystems are indicated. Here the RedWater drill is carried by NASA's ATHLETE rover.

A Rodwell system dubbed “RedWater” is under design specifically for use in the martian environment by Honeybee Robotics.^24,53^ This system is capable of mining water at up to 25 m depth and through up to 20 m of overburden. RedWater implements two proven terrestrial technologies: coiled tubing (CT) for drilling and Rodwell for water extraction. The RedWater components are shown in *Figure 3*. The end of the CT tube has a Bottom Hole Assembly (BHA), which is a motor and a drill bit for drilling into the subsurface. To remove chips, compressed martian air is pumped down the tube.

Once the hole is made, the CT is left in the hole and used as a conduit for water extraction. The BHA contains a rotary-percussive drill subsystem similar to the one used in Honeybee Robotics Deep Drill^54^ and heaters. On reaching an ice layer, the drill continues for another ∼3 m and then stops advancing forward, but the bit continues spinning. The drill deploys a flexible packer to seal off the hole and subsequently, the heaters are turned on to melt the surrounding ice. After melting a section of ice, the borehole is further pressurized, and a valve opens to allow water to flow to the surface tank, as in terrestrial geysers.

**Fig. 4.**
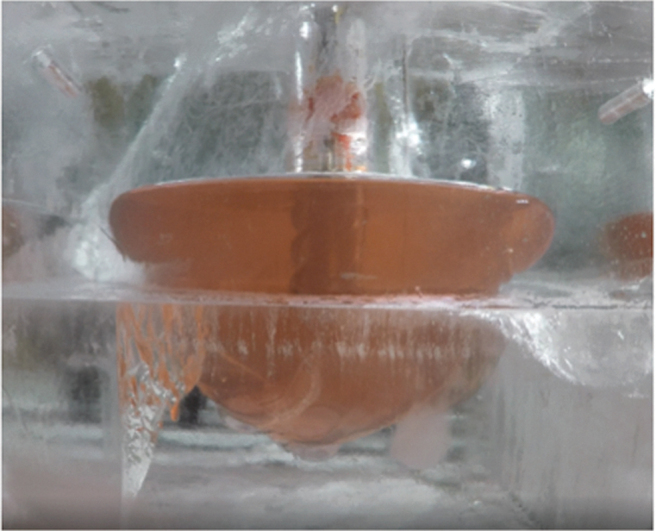
RedWater undergoing testing in a block of water ice at −25°C. Shown is a melt pool formed after ∼2 h of melting while the auger bit (*center*) is being rotated to stir up the water. The pool is 10″ in diameter and contains 15 L of water.

A prototype RedWater system was designed and tested in a −25°C block of ice at the same temperature (*Fig. 4*). The 15 kg melt pool was created in ∼2 h by using ∼1 kW heater system (which implies thermal efficiency of ∼50%). Extrapolating to mass production, 1 metric ton of water could be produced in 10 days.

Beyond a Rodwell, alternate methods of acquiring water from subsurface ice include strip mining and/or the use of explosives. For strip mining, machinery mechanically removes the dry overburden and then a jackhammer, drilling, or sawing machine breaks apart the subsurface ice. This technique is a form of surface mining where the overlying material is removed to expose the resource to be harvested. Another option is to use explosives that can be detonated in a controlled manner to remove the unwanted overburden deposit and simultaneously expose and break apart the desired subsurface ice.

Regardless of the ice extraction technique ultimately chosen, a backup technique should be available given the criticality of harvesting ice. In designing the overall architecture for the initial Mars base, equipment associated with different excavation techniques may have multiple functions. For example, a trenching machine for digging ice may also be used for digging pipelines for conveying liquids and gases throughout the base. Multiple use cases for equipment should be considered when designing contingency plans for ice extraction capabilities.

#### (3) Water distribution system

The ice harvested from the mine site will likely require transport from the mine to closer to the human base for additional processing and ultimate use. Water and/or ice can be transported via multiple means, for example, pipelines, pump systems, hose systems, and/or truck delivery. Architecture trades will be required to determine the most efficient and effective means of resource transport. A key consideration will be the volume of water to be transported. For example, a pipeline system may not be needed initially if the volumes of water are relatively low.

In this scenario, trucks may be sufficient to transport the water resource. However, to establish a permanent base, a pipe system to the resource is optimal since the free flow of water stimulates the growth of civilization. A proper regard for the importance of gravity in influencing water flow direction is important. It is most advantageous if the water consumer is located at a lower elevation than the water supply. If this is not the case, then the energy demand required for pumping water from the storage location to the consumer must be assessed. Fluctuation of ambient temperature is also a significant design factor on Mars. There is much terrestrial technology that can contribute to understanding low-temperature installation, as there are numerous major municipal water supply districts in extremely cold locations on the Earth such as Canada, Russia, and Scandinavia.

The type of resource transportation system will also be dependent on the distance between the mining site and the human base and Starship vehicles. This distance will not be known until final site selection and master planning the design of the Mars base. When developing the resource transport system, the number of resource transfers should be minimized since any transfer of materials introduces inefficiencies in the system.

Locations of processing and storage facilities are also important considerations when planning a water distribution system. For example, the system for transferring water from the mine site to the base may, in part, be dependent on whether the water purification and/or additional processing is done at the mine site or at the human base. This decision is architecture-dependent but will influence the amount and type of infrastructure available at both locations.

The method of water storage is also important. Trades are required to optimize the design of the transfer process depending on whether water will be stored as ice and melted as needed or if large tanks of liquid will be maintained for on-demand use. Such decisions may be driven by the selection of the primary water extraction technique. For example, continuous operations of a Rodwell system require significant energy to maintain the underground pool of liquid water, so one possibility is extracting all the water needed from a Rodwell (or multiple Rodwells) to maintain the base for a significant amount of time at once, and then storing that water for future use. Regardless, growth and maintenance must be considered when designing each asset and component of the martian water distribution system.

#### (4) Water/ice purification

Water extracted from *in situ* sources on Mars must be tested for contaminants and potential biological presence and purified accordingly.^55^ The level of tolerable impurities must be identified for different uses of the water resource, for example, requirements for propellant purity will differ from requirements for drinking water. However, the fraction of water required to sustain the human presence on Mars will be relatively small compared with the water required to refill the Starship vehicles. As is routine on the ISS, 90% of water can be recycled; this would also be the case on Mars.

The first step in the water purification process is a laboratory analysis to characterize the impurities and guide the treatment processes. System reliability must be high for this testing, as chemical or biological contamination may significantly affect the health of the crew.^56–59^ In the design of water analysis technology for Mars, avoidance of consumables is ideal to minimize the types and amounts of specific materials that must be transported from the Earth. *A priori* knowledge of ice characteristics will help inform the design of a suitable water analysis system.

There are multiple methodologies that can be employed for water and/or ice purification. Physical treatment technologies utilize phase separation of constituents in water through density differential (flotation, settling, centrifugation), phase changes (distillation, evaporation, freezing), selective physical barrier (filtration), reverse osmosis, or differential surface chemistry (capillary and chromatographic separation, ion exchange, sorption).^55,60^ For these methodologies, chemical consumables are generally not utilized but maintenance and replacement of hardware components is often required.

Chemical treatment options can remove unwanted contaminants as well as disinfect a water supply. These methods are generally rapid and reliable, although they typically consume reactants that must be resupplied and can produce byproducts that must be considered for waste management. Mars missions should prioritize chemical treatment technologies where reactants can be produced *in situ* and the byproducts are benign.^55^ Numerous chemical purification methods exist, each with advantages and disadvantages as well as varying applicability to a long-duration space flight.^55^

Biologic treatment options can also be considered to determine the optimal combination of techniques for water purification on Mars.^55,61,62^ Although the kinetics of biological processes are typically lower (hours to days) compared with physical or chemical processes (seconds to minutes), biological processes are capable of auto-regeneration and may provide a sustainable purification system within a bioreactor on Mars. Bioreactors have become common practice on the Earth^63^ and can consist of aerobic, anaerobic, or phototrophic systems. Trade studies must be conducted to determine the optimal combination of physical, chemical, and/or biologic water purification systems to support the Mars base.

#### (5) Distribution, storage, and use of water

Once water has been extracted from the martian subsurface, it must be distributed to end users or stored until needed. On Mars, water can be stored in either the frozen (ice) or liquid (water) state. The most efficient means for distributing water will greatly depend on the state in which it is stored. Trade studies should be conducted to determine the most efficient means for storing the water. Some considerations are outlined here.

If a Rodwell system is used continuously to pump liquid water from the martian subsurface, then the water will be extracted and may be stored as a liquid. For safety, the base should always maintain some liquid water for crew use. Depending on production rates, some water would likely be stored for longer periods of time possibly as ice, which would remain frozen in the ambient martian environment but would need to be protected against sublimation.

The option of frozen storage may apply to the production of propellant for Starship return trips to the Earth, which occur only every 2 years. Trade studies are required regarding the options to constantly produce propellant at the base to minimize any surges in power needs versus producing propellant on demand in a batch process. Batch processing would reduce the cryogenic cooling requirements for longer term storage but would likely be difficult due to the power requirements to generate propellant all at once. Propellant production also requires CO_2_ and its collection and processing must be integrated with the associated water production.

Once martian water is extracted, further processing requires a combination of electrolysis and the methanation process (*e.g.*, Sabatier).^60^ Electrolysis converts four water molecules into four H_2_ and two O_2_ molecules (4H_2_O → 4H_2_ + 2O_2_). Methanation uses a catalyst to convert carbon dioxide from the atmosphere with hydrogen from the water electrolysis to produce methane and water (CO_2_ + H_2_ → catalyst → CH_4_ and H_2_O).

The oxygen propellant is generated by the water electrolysis process. The oxygen and methane are liquefied and stored as cryogens due to practical considerations of total volume of the propellants in gas versus liquid phase. These products must then be stored until ready for use.

Many of the activities described for martian water ice ISRU can be conducted both before human arrival and post-human arrival on Mars. Initial uncrewed flights should be used to prove out high-risk and high cycle items as early as possible. For example, trucks can move autonomously across remote terrain to transport machinery to the mine site. However, one may not want or need to fully deploy the full excavation and ISRU systems unless increased automation is possible. Typically, such complex deployments become easier with human intervention.

However, some subcomponents of the ISRU system may be tested before human arrival. For example, the ISRU electrolysis and methanation plant could operate with fresh water brought from the Earth in conjunction with CO_2_ acquired from the martian atmosphere. Such an activity would be decoupled from the more complex ice excavation system and could operate for 1 or 2 years before human arrival.

During this time, it would demonstrate the long-term, high cycle operation of the CO_2_ compressor, methanation system and catalyst, and products purity. On-site tests of propellant tank insulation could also be conducted to test different methods of insulation. Performance of components common to multiple systems (*e.g.*, drives, motors) needed to perform for long periods of time can be tested to measure reliability. It is relatively easy for a human to perform maintenance activities such as changing out end effectors for drills and other hardware components, whereas such maintenance can be difficult to automate.

Therefore, long-duration performance testing hardware should be conducted before crews arrive, whereas more complex tasks should take advantage of the availability of human problem-solving skills once they arrive. However, in terms of enabling martian ISRU for support of the human base, the highest priority activity for the first uncrewed Starship missions is to characterize the Mars ice resource to finalize designs of the water extraction, purification, and processing systems for ISRU and begin testing out some generic high cycle systems in the martian environment.

## Additional High Priority Starship Objectives

In addition to ISRU considerations, we outline additional high-priority objectives for the first two uncrewed Starship missions to optimize development of the human base on Mars. These tasks include deploying initial power infrastructure elements, characterizing the local environment at the landing site, implementing initial radiation shielding, food growth experiments, autonomous regolith excavation and drilling demonstrations, pre-positioning of supplies, and assessing hazards to human habitability during surface operations.

### Deploy Initial Power Infrastructure

The successful deployment and operation of a power infrastructure is of highest priority for both uncrewed and crewed missions to the martian surface. Since the availability of power is critical to surface operations, an initial deployment and test of power systems is a priority for the first Starship vehicles that land on Mars. For the landed missions (both with and without crew), power will initially be provided by battery power, followed by solar arrays once they are deployed and verified. A backup power source is also required that can be obtained via battery, chemical, and/or propellant sources.

Solar power is likely an early power option beyond just batteries, but with the regular occurrence of global dust storms, nuclear power will be the long-term solution. Nuclear reactors can, thus, also be used for early crews if available. Although initial demands may be smaller, Starship's payload capacity will allow deployment of very large nuclear power facilities. As an example, the Hyperion reactor design weighs 20 metric tons and produces 25 MW of power.

Significant uses of power include propellant generation and storage on the martian surface, Starship vehicle support, storage of water and food, and support for crew extravehicular activities away from the main base. Power will likely be allocated in a distributed manner with a number of networked assets that can access and harness the required power where and when needed. Contingency power systems are also required to ensure stay-alive power levels. If needed, non-essential activities such as propellant production and resource exploration activities could be curtailed in emergencies.

A key technology demonstration that could be conducted on the first uncrewed Starship missions is the autonomous deployment and operation of a solar power system. Deployment methodologies as well as solar panels and array designs should be tested. For example, solar panels on space missions are typically optimized for high performance. Trades in solar panel design and manufacture for lower performance may be offset by simply deploying a larger array on Mars. For example, terrestrial solar panels are often mounted on heavy support structures that track the Sun to increase power production over time.

The sheer areal extent of solar arrays required on Mars may prohibit the use of tracking devices, and the first missions can assess the merits and drawbacks of deploying the arrays on the ground or above ground, with or without tracking capabilities. For example, simpler deployment techniques such as pressurizing ribs to uncoil large rolls of solar arrays should be tested. In addition, maintenance of the arrays (including dust removal) must be tested to determine optimal long-term solutions.

### Environmental Characterization

The first uncrewed Starships provide an unprecedented opportunity to collect environmental data at the landing site which will be valuable for the design and construction of future equipment and infrastructure to be flown to Mars. A basic weather station to measure atmospheric and surface temperatures, wind speed and direction, atmospheric dust content, atmospheric pressure, and radiation levels is necessary to minimize uncertainty regarding the environmental conditions expected for future missions. Surface properties such as trafficability, geomorphology, and geotechnical properties of the surrounding landscape should be assessed to optimize future infrastructure development such as roads, landing pads, and habitats.

### Test High-Risk Items

The flight of two uncrewed Starship vehicles to the martian surface also provides the opportunity to test several high-risk items before human arrival. Operations that may be particularly sensitive to unknowns in the martian environment and require long duration testing have the highest priority on the first Starship missions.

### Radiation Shielding

Radiation shielding is a high-priority requirement to ensure human safety on the surface. Astronauts will require adequate radiation shielding while working and living within the Starship vehicle on Mars and will also require radiation mitigation when working outside of the Starship. For example, outdoor protected areas to conduct maintenance activities, laboratory work, and for transit between different Starships and/or facilities are required.

Limited measurements of radiation levels on the martian surface are available^64,65^ along with numerous radiation models to predict expected radiation levels.^66,67^ However, existing surface radiation measurements on Mars are at discrete locations not coincident with the expected landing sites of Starship and, thus, the applicability of these measurements to different locations is unknown. Radiation models also include multiple assumptions and uncertainties and must be validated with surface measurements at the Starship landing site. Therefore, a long-duration radiation monitoring station on the surface at the Starship landing site along with different materials and designs for radiation shielding testing are envisioned, including the potential use of local Mars materials for radiation shielding purposes.

### Food Production

Food growth experiments are an opportunistic activity to conduct on early Starship landers. The ability for humans to grow their own food on Mars will be key to becoming self-sustaining.^68^ Food production and processing is highly dependent on environmental conditions (*e.g.*, reduced gravity, temperature, radiation, soil chemistry, etc.), which are variable between sites.^69–71^ Plant growth experimentation is necessary to assess the effects of various conditions such as high carbon dioxide levels, low light, low water and nutrient levels, pollination efficiency, and the effect of low pressure and magnetic fields on plant growth and crop productivity.^68,72–75^

Most of these martian conditions are not believed to be limiting for crop productivity and can be compensated for within a greenhouse environment.^68,76,77^ Hydroponics will also be assessed on Mars, building on hydroponic food production research on the ISS with the Vegetable Production System (VEGGIE).^78^ In addition, fresh plants and crops are expected to have a positive impact on the psychological health of the crew.^75^ Food production can be tested to a limited extent on robotic missions, whereas human-tended plant growth activities can potentially be conducted with less operational complexity.

### Autonomous Construction Operations

Additional experiments to be considered for the first uncrewed Starship landers are centered on the processes of excavation, drilling, and building by using autonomous operations. Excavation and drilling are key activities to enable long-term survival of humans on Mars. These activities are critical for ISRU as well as civil engineering applications such as building berms, roads, trenches, and landing pads.^79–81^

Much of this work can be done robotically, and the first Starship missions can be utilized to test and demonstrate autonomous techniques. These initial construction activities will inform variations on future designs for equipment to be flown to Mars, and to develop as much infrastructure as feasible before the first human landings.

### Pre-position Supplies Before Human Landings

The substantial cargo capacity (100 metric tons) of the Starship vehicle provides ample opportunity to deliver and pre-position key supplies on Mars in advance of human arrival. The Starship vehicles themselves will serve as initial infrastructure at the Mars base. The large interior volume of Starship (both pressurized and unpressurized) provides significant space to serve as shelter in terms of habitation quarters, laboratory and work space for humans, and storage space for equipment and supplies.

The first humans to Mars should be able to live within the Starship vehicles that flew them from Earth. However, in the event of any off-nominal issues, the human crew also has the original uncrewed Starship vehicles that first arrived on Mars as backup sources of habitation and storage space as well as spare parts and supplies.

The first uncrewed Starships can also pre-position stockpiles of food and water on Mars before human arrival. This approach will provide redundancy and ensure adequate supplies to support the humans on the martian surface, as the crewed Starships will also bring food and water supplies. Food is key to human sustenance, and water will be important not only for human use and consumption but also to enable ISRU on Mars. In the event that water cannot be harvested from Mars in the time or quantities required to support ISRU activities, contingency rescue scenarios, including delivery of water from Earth via Starship, can be used to save the crew if needed.

Communication systems from the first uncrewed Starships will be prepositioned and tested before human arrival on Mars. These systems will be established and tested for communication between Earth and the uncrewed Starships on Mars. Successful communication is required to ensure that the humans will have the ability to communicate with the Earth, even in the event of any off-nominal issues with the communications system in the human-tended Starships.

The first uncrewed Starship will also preposition additional equipment such as rovers to enable mobility of equipment and humans. Rovers should ideally be equipped with a modular ability to complete multiple tasks and maintain energy efficiency. Rovers conceivably would be used for such varied tasks as deployment of solar arrays, excavation of regolith for construction and ISRU, moving cargo and material to different areas of the base site, and transport of equipment to field sites. Depending on the Starship landing site locations and the size of the human base, rovers may be needed to transport people and/or cargo between Starships and more remote locations (such as field science sites, ISRU sites for ice excavation, etc).

### Landing Site Preparation

Early Starships will land on native martian terrain with no landing site preparation available; however, operational capabilities can be enhanced for subsequent flights through the development of dedicated landing areas. Landing pads must not only withstand the heat and pressure of the engine thrusters, but also berms surrounding the pads must mitigate regolith sandblasting of the surrounding area.^82^

These protective measures are important to allow buildup of a human base with infrastructure in close proximity to the pads. The construction of landing pads will also prevent lander exhaust plumes from excavating holes under the lander, possibly melting subsurface ice and/or tipping the lander and causing lander damage.^83^ Multiple surface stabilization techniques have been proposed for the construction and evaluation of landing pads such as use of *in situ* materials, 3D printing, and freezing of landing areas.^82,84,85^ Multiple options could be assessed on Mars by producing multiple “landing coupons” using equipment delivered in the first uncrewed Starship and testing under subsequent uncrewed Starship landings.

### Human Habitability and Astrobiology

To enhance human health and safety, early uncrewed Starships could also include a payload(s) to assess the hazard potential to operations on the surface, including characterizing materials that humans are likely to encounter for potential biological, chemical, and mechanical hazards. This would include characterization of the water ice and dust along with refining our understanding of perchlorates in the soil. Instruments that could characterize these aspects of the martian environment and evaluate the biological potential in the area of interest would help inform mitigation measures and risk evaluation for initial crews traveling to Mars.

Regarding the potential for life on Mars, recent work has examined the issue of martian habitability, which suggests that Mars could have harbored life within the past 5 million years.^86^ Near-surface ice likely had water activity levels high enough to support life at higher obliquities, and ground ice is an especially efficient means for preserving organic biomarkers for extended periods of time.^87–89^ Multiple energy sources to support martian life exist in the form of carbon dioxide and nitrogen in the atmosphere, nitrates in the soil, and/or perchlorate combined with basaltic rock.^86^ This combination of parameters (water, energy, nutrients) leads to a reasonable possibility of extinct and/or extant life on Mars, which cannot be ruled out at the present time.

The instrumentation for biological hazard assessments could, thus, also provide additional scientific data in the field of astrobiology to help determine whether life ever arose on the Red Planet, a fundamental scientific question driving the exploration of Mars.^86,90–94^ These investigations can provide baseline measurements of the biologic potential for native martian life in the near-surface environment on the uncrewed missions in preparation for the much more thorough search for life studies that will be enabled by having astronauts on the surface to conduct more sophisticated experiments and access subsurface environments.

## Conclusions and Future Vision

The SpaceX Starship vehicle fundamentally changes the paradigm for human exploration of space and enables humans to develop into a multi-planet species.^1–3^ The first steps of developing a self-sustaining human civilization on Mars utilizing planned SpaceX missions to the Red Planet have been outlined. The first Starships flown to Mars will be uncrewed and provide unprecedented opportunities to each deliver ∼100 metric tons of cargo and conduct precursor work to enable martian ISRU for a sustained and self-reliant human presence.^3^

Highest priority activities for the uncrewed Starships will be pre-emplacement of supplies and infrastructure, testing of key technologies, and conducting resource prospecting to map and characterize water ice.

Starship missions to the lunar surface may be an important stepping stone for reaching Mars both technically and programmatically. The Moon can be a testbed and demonstration platform for ISRU technologies as well as Starship operations. Regardless of destination, this article highlights the importance of early master planning to enable development of a sustained human presence on another world.

ISRU is a key component for enabling humans to decrease reliance on the Earth and instead develop a self-sustaining presence off-planet. The development and expansion of an initial Mars base will allow more humans to live on the Red Planet, and thereby accomplish the historic and unprecedented task of making humanity a multi-planet species.
